# Supplementary material for: High Seroprevalence to Aedes-borne arboviruses in Ethiopia: a Cross-sectional Survey in 2024
Source: medRxiv. 2024 Sep 26:2024.09.19.24313917. Preprint. [Version 2] doi: 10.1101/2024.09.19.24313917 (PMC11451662; doi:10.1101/2024.09.19.24313917)
Supplement: Supplement 1 [file media-1.pdf]

SUPPLEMENTAL MATERIALS

Supplemental Figure 1: Age specific seroprevalence by arbovirus and for IgG (top panel) and IgM (bottom panel). Bar size is the proportion who tested positive ("reactive") by arbovirus. The

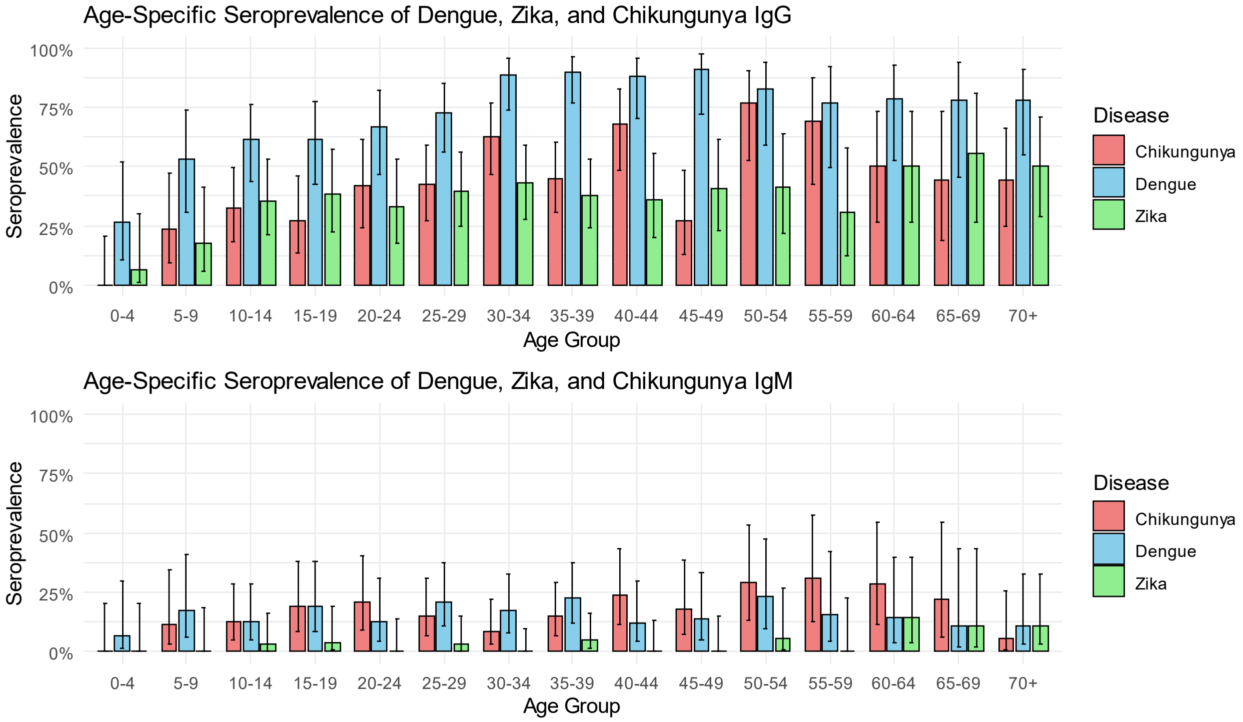

corresponding whiskers are Wilson score confidence intervals.

Age specific seropositivity for dengue IgG was high across most age groups, beginning at around 25% in the 0 to 4 age group and reaching a high of approximately 90% in the 30 – 34 age group. Chikungunya IgG seropositivity was next highest, reaching a high of 75% in the 50 – 54 age group. As with both dengue and chikungunya, Zika IgG seropositivity was lowest in the 0 – 4 age group, increasing with age but leveling off around age 15 – 25. All age groups exhibited IgG seropositivity to all three viruses.

IgM to dengue virus and chikungunya virus was higher than for Zika virus, though all three were present in almost all age groups (with an exception of Zika IgM being absent in the 0 – 4, 20 – 24,

30 – 34, and 40 – 49 age groups). Dengue IgM seropositivity was dominant in age groups up until the 40 – 45 age group, when chikungunya IgM seropositivity became highest.

Supplemental Figure 2:

IgG responses for ZIKV and DENV by test status. Since all ZIKV IgG positives were also DENV IgG positive, there is no way to look at the IgG among ZIKV IgG positives but DENV IgG negative (panel A). Panel B shows, for all individuals who were ZIKV IgG negative, the differences in quantitative ZIKV IgG response among those who were DENG IgG positive (blue/green) and those who were DENG IgG negative (orange). Panel C shows, for those who were DENG IgG positive, the difference in quantitative DENG IgG values for those who were ZIKV IgG positive or negative. DENV IgG was much higher among individuals who tested positive for ZIKV IgG (panel C). ZIKV IgG is also higher among those who tested positive for DENV IgG (panel B), but the difference is small in comparison to association between

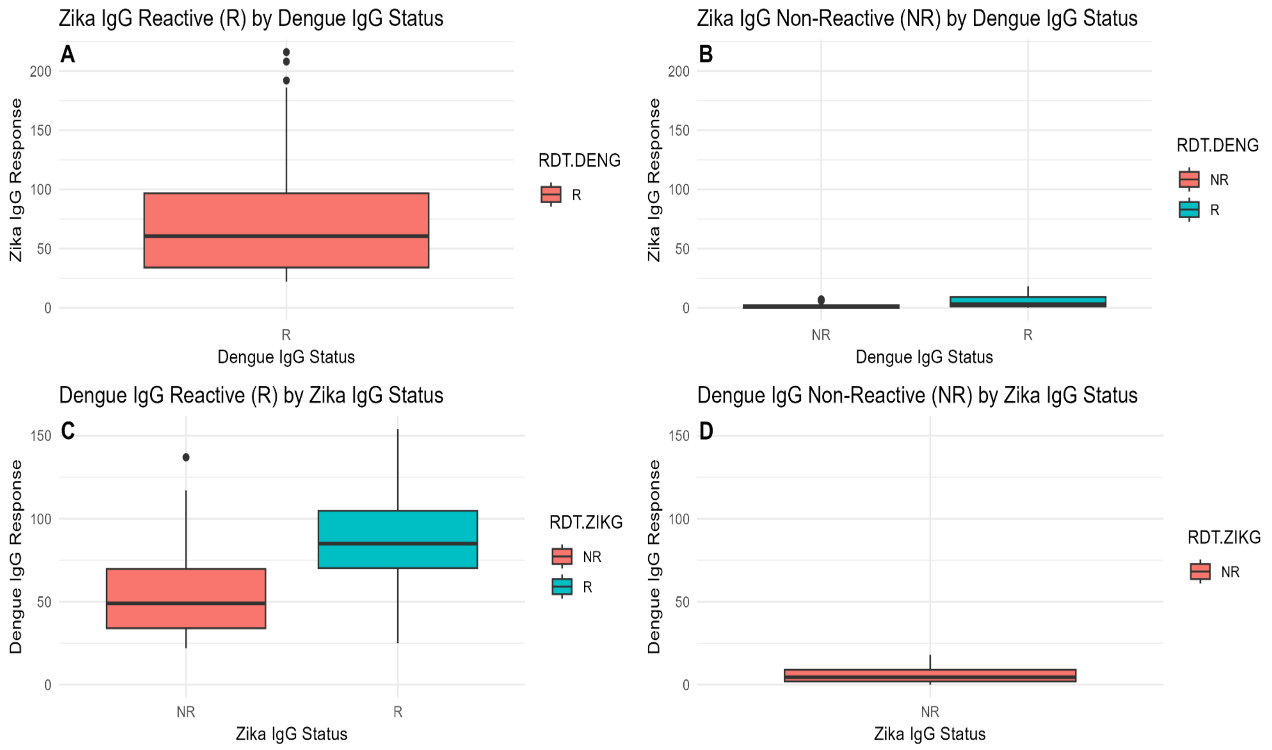

Supplemental Table 1: Linear mixed models (random intercept for household) for IgG. The "Model" column indicates which of the three viruses was the outcome while the "Predictor" column indicates the virus IgG measures that were used as predictors for in that model.

Model	Predictor	Estimate	CI	Std.Error	p-value
Dengue IgG	(Intercept)	32.10	(27.47, 36.73)	2.36	<0.0001
Dengue IgG	Zika IgG	0.52	(0.45, 0.59)	0.04	<0.0001
Dengue IgG	Chikungunya IgG	0.14	(0.09, 0.18)	0.02	<0.0001
Zika IgG	(Intercept)	-5.61	(-12.11, 0.89)	3.31	0.0915
Zika IgG	Dengue IgG	0.72	(0.62, 0.82)	0.05	<0.0001
Zika IgG	Chikungunya IgG	-0.07	(-0.13, -0.02)	0.03	0.0087
Chikungunya IgG	(Intercept)	23.85	(11.20, 36.50)	6.46	0.0003
Chikungunya IgG	Zika IgG	0.70	(0.47, 0.93)	0.12	<0.0001
Chikungunya IgG	Dengue IgG	-0.27	(-0.48, -0.07)	0.10	0.0084

Supplemental Table 2: Linear mixed models (random intercept for household) for IgM. The "Model" column indicates which of the three viruses was the outcome while the "Predictor" column indicates the virus IgG measures that were used as predictors for in that model.

Model	Predictor	Estimate	CI	Std.Error	p-value
Dengue IgM	(Intercept)	7.99	(6.52, 9.47)	0.75	<0.0001
Dengue IgM	Zika IgM	0.33	(0.19, 0.47)	0.07	<0.0001
Dengue IgM	Chikungunya IgM	0.39	(0.29, 0.49)	0.05	<0.0001
Dengue IgM	ZIKa IgG	0.06	(0.04, 0.09)	0.01	<0.0001
Dengue IgM	Chikungunya IgG	-0.06	(-0.08, -0.03)	0.01	<0.0001
Zika IgM	(Intercept)	0.09	(-1.39, 1.57)	0.76	0.9050
Zika IgM	Dengue IgM	0.24	(0.16, 0.32)	0.04	<0.0001
Zika IgM	Chikungunya IgM	0.18	(0.10, 0.26)	0.04	<0.0001
Zika IgM	Dengue IgG	0.01	(-0.01, 0.04)	0.01	0.2430
Zika IgM	Chikungunya IgG	-0.03	(-0.05, -0.01)	0.01	0.0013
Chikungunya IgM	(Intercept)	3.43	(0.39, 6.47)	1.55	0.0281
Chikungunya IgM	Dengue IgM	0.42	(0.27, 0.58)	0.08	<0.0001
Chikungunya IgM	Zika IgM	0.41	(0.20, 0.62)	0.11	0.0002
Chikungunya IgM	Dengue IgG	0.06	(0.01, 0.12)	0.03	0.0178
Chikungunya IgM	Zika IgG	-0.08	(-0.12, -0.03)	0.02	0.0013
